# Peptidergic signaling from clock neurons regulates reproductive dormancy in *Drosophila melanogaster*

**DOI:** 10.1371/journal.pgen.1008158

**Published:** 2019-06-13

**Authors:** Dóra Nagy, Paola Cusumano, Gabriele Andreatta, Ane Martin Anduaga, Christiane Hermann-Luibl, Nils Reinhard, João Gesto, Christian Wegener, Gabriella Mazzotta, Ezio Rosato, Charalambos P. Kyriacou, Charlotte Helfrich-Förster, Rodolfo Costa

**Affiliations:** 1 Department of Biology, University of Padova, Padova, Italy; 2 Department of Genetics and Genome Biology, University of Leicester, Leicester, United Kingdom; 3 Neurobiology and Genetics, Theodor-Boveri-Institute, Biocenter, University of Würzburg, Würzburg, Germany; Washington University in Saint Louis School of Medicine, UNITED STATES

## Abstract

With the approach of winter, many insects switch to an alternative protective developmental program called diapause. *Drosophila melanogaster* females overwinter as adults by inducing a reproductive arrest that is characterized by inhibition of ovarian development at previtellogenic stages. The insulin producing cells (IPCs) are key regulators of this process, since they produce and release insulin-like peptides that act as diapause-antagonizing hormones. Here we show that in *D*. *melanogaster* two neuropeptides, Pigment Dispersing Factor (PDF) and short Neuropeptide F (sNPF) inhibit reproductive arrest, likely through modulation of the IPCs. In particular, genetic manipulations of the PDF-expressing neurons, which include the sNPF-producing small ventral Lateral Neurons (s-LN_v_s), modulated the levels of reproductive dormancy, suggesting the involvement of both neuropeptides. We expressed a genetically encoded cAMP sensor in the IPCs and challenged brain explants with synthetic PDF and sNPF. Bath applications of both neuropeptides increased cAMP levels in the IPCs, even more so when they were applied together, suggesting a synergistic effect. Bath application of sNPF additionally increased Ca^2+^ levels in the IPCs. Our results indicate that PDF and sNPF inhibit reproductive dormancy by maintaining the IPCs in an active state.

## Author summary

Diapause is a hormonally mediated process that allows insects to predict and respond to unfavourable conditions by altering their metabolism and behavior to resist the oncoming environmental challenges. In *Drosophila melanogaster* females a protective state of reproductive dormancy is induced by lower temperatures and shorter photoperiods that mimic the approach of winter. By genetically manipulating the circadian pacemaker s-LN_v_s cells, which express two neuropeptides, Pigment dispersing factor (PDF) and short Neuropeptide F (sNPF), we were able to modulate levels of gonadal arrest. PDF and sNPF appear to act as antagonists to dormancy, as do the Drosophila insulin-like peptides (dILPs) that are expressed in the insulin producing cells (IPCs). Indeed, we observe that the axonal projections from the s-LNvs appear to overlap with those from the IPCs implying that the clock cells signal to the IPCs. We confirm this possible communication by applying the two synthetic peptides to the IPCs and detecting a response in the IPC signal transduction pathway. We conclude that the clock neurons activate the IPCs via PDF and sNPF, which in turn release the dILPs, antagonise dormancy and lead to reproductive growth, thereby uncovering a neurogenetic circadian-overwintering axis.

## Introduction

To synchronize with the Earth’s rhythmic environment, most higher organisms have evolved endogenous time-keeping systems [1,2]. While the highly conserved circadian clock is well-characterized [3,4], our knowledge of the seasonal clock that governs the overwintering response (diapause) in insects is still fragmentary [5,6]. Diapause refers to an alternative developmental program, typically induced by the shortening days and falling temperatures of the approaching winter [1,7]. Diapausing animals are characterized by low metabolic rate, drastically decreased food intake, extended lifespan, and increased stress resistance [8–15]. The fruit fly *Drosophila melanogaster* exhibits an adult reproductive ‘diapause’ manifested by arrested ovarian development which is stimulated by low temperatures and can be enhanced by short photoperiods [15,16]. While the *Drosophila* literature refers to this phenomenon as ‘diapause’ rather than ‘dormancy’ or ‘overwintering’, we recognize that it is not a classic photoperiodically-induced state because it requires cold-temperature to induce the reproductive quiescence. Nevertheless, it shows features that are commonly associated with responses that are resistant to environmental stresses [7,15,16].

An increasing body of evidence suggests that insulin-like signaling is a key regulator of diapause in numerous species [13,17–20]. In *Drosophila*, 4 of the 8 identified insulin-like proteins (DILP1, -2, -3, -5) are produced in 14 median neurosecretory cells (designated as Insulin Producing Cells, IPCs) of the *Pars intercerebralis* (PI), which are anatomically connected to the key neuroendocrine system that governs hormonal regulation of gonadal arrest [21–25]. Even though the center for dormancy control is believed to be in the PI [26–28], the neurosecretory cells in this brain area, including the IPCs, do not have a circadian clock, and would therefore have to receive any timing information (if any) from other cells [29–33]. A challenging question is how the environmental signals that trigger dormancy (i.e. decreasing photoperiod and temperature) including putative timing information, are perceived, interpreted, and converted into hormonal signals in the brain, leading to the overwintering phenotype [34].

Even though several neuropeptides, neurotransmitters, and peptide hormones have already been identified as modulators of function of the IPCs (reviewed in [35,36]), there are still gaps in our understanding of how the activity of these cells is controlled. Recent research revealed a synaptic connection between the IPCs and one group of dorsal clock neurons (DN1), raising the possibility of a direct modulatory effect exerted by the circadian system [33]. Indeed, natural variants of the *timeless* clock gene in *D*. *melanogaster*, namely the *s-tim* and *ls-tim* allelic variants, are known to have a dramatic effect on the inducibility of reproductive dormancy [37,38].

In our study, we primarily focused on neuronal clusters that, based on their axonal projections to the dorsal brain (dorsal protocerebrum), could play an intermediary role in conveying dormancy-inducing signals towards the IPCs. Neurites of the small ventral lateral neurons (s-LN_v_s) project to the dorsal protocerebrum, where they rhythmically release the circadian neuromodulator PIGMENT DISPERSING FACTOR (PDF) [39]. PDF is also expressed in the large ventral lateral neurons (l-LN_v_s), and in two groups of non-clock cells, a developmentally transient neuronal cluster in the Tritocerebrum (designated as PDF-Tri), and a small number of neurons in the eighth abdominal neuromere of the ventral ganglion (designated as PDFAb neurons) [40]. PDF is a key coordinator of pacemaker interactions and behavioral rhythms [41–43], of sleep and arousal [44,45] and of sexual behavior [46]. PDF may also be involved in diapause regulation in the blow fly *Protophormia terraenovae* [47], the mosquito *Culex pipiens* [48], and the bean bug *Riptortus pedestris* [49]. However, its effects appear to be contradictory and the mechanisms through which PDF acts on diapause are unclear.

Short Neuropeptide F (sNPF) has been implicated in the modulation of diapause in the Colorado potato beetle [50] and has been shown to stimulate ovarian development in the locust [51,52]. In *Drosophila* sNPF increases food intake and body size [53] and enhances growth [54]. This peptide is broadly produced in the *Drosophila* nervous system [53,55], including the s-LN_v_s [56]. A small set of bilaterally symmetric neurons in the *Pars lateralis* (PL), defined as dorsal-lateral peptidergic neurons (DLPs), also express sNPF. DLPs have axon terminations in the proximity of the IPCs, and co-express the multi-functional neuropeptide Corazonin (Crz) [57], which has also been proposed as a diapause regulating peptide in the hawkmoth *Manduca sexta* [58].

The G protein-coupled receptors for PDF, sNPF, and Crz (PDFR, sNPFR1, and CrzR, respectively) have already been characterized and extensively studied in *Drosophila* [57,59–67]. Interestingly, sNPFR1 and CrzR have been found to influence the activity of the IPCs [53,54,57,68]. However, to date no studies have reported PDF signaling to the IPCs.

In the present study, we demonstrate that PDF and sNPF produced by PDF-positive (PDF^+^) neurons, reduce the induction of dormancy in *Drosophila*, mainly through a direct effect on the IPCs. Conversely, the Corazonin-expressing DLP neurons do not seem to be involved. Using live imaging, we show that the IPCs respond to both PDF and sNPF peptides with increasing levels of cAMP and to sNPF additionally with increasing Ca^2+^ levels suggesting that PDF and sNPF positively modulate IPCs activity and thereby inhibit gonadal arrest.

## Results

### Genetic manipulations of PDF^+^ neurons alter dormancy levels

**Fig 1:**
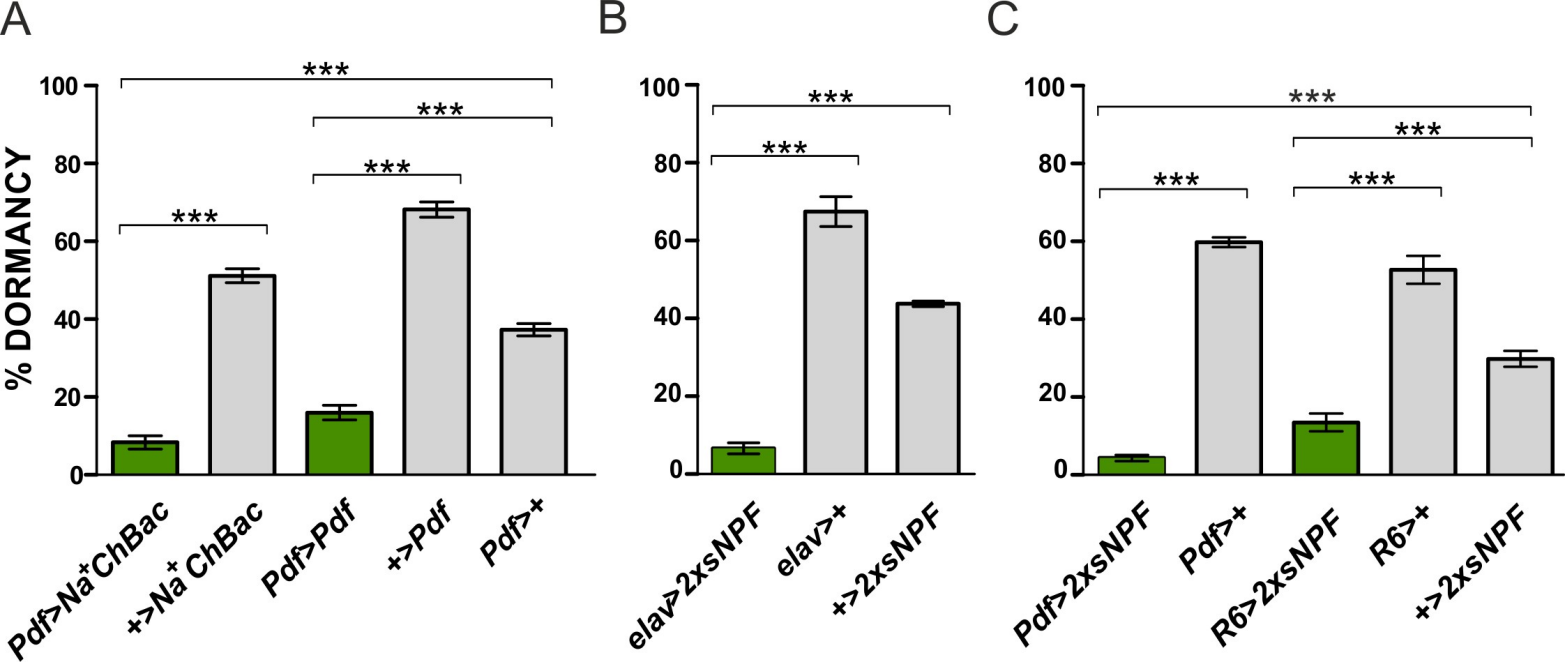
Enhanced activity of PDF-expressing neurons reduces dormancy levels. (A) Hypersensitization of PDF^+^ neurons (*Pdf*>*Na*^*+*^*ChBac*) and overexpression of PDF (*Pdf*>*Pdf*) both result in a significant reduction of reproductive arrest. (B) Pan-neuronal overexpression of sNPF (*elav*>*2xsNPF*) leads to a significant decrease in the proportion of arrested females. (C) Overexpression of sNPF in the PDF-expressing neurons (*Pdf*>*2xsNPF*) and in the small ventrolateral neurons (*R6*>*2xsNPF*) triggers females to exit from dormancy. Numbers within bars refer to the number of dissected females considered in the dormancy assays. Data are presented as mean ± SEM. ANOVA on arcsine transformations, followed by *post-hoc* Tukey HSD test. ***p<0.001, n.s. not significant.

To examine whether the PDF-expressing neurons have a role in the regulation of reproductive arrest, we used a *Pdf*-Gal4 driver to target gene expression specifically in PDF^+^ neurons including both s- and l-LN_v_s [39,41]. First, a bacterial depolarization-activated sodium channel (Na^+^ChBac) was expressed in these neurons (*Pdf*>*Na*^*+*^*ChBac*). Such manipulation will enhance the release of neurotransmitters and neuropeptides, including PDF and sNPF [69]. *Pdf*>*Na*^*+*^*ChBac* flies showed significantly lower levels of dormancy compared to controls (p<0.001; Fig 1A). Importantly, Na^+^ChBac expressing and control flies shared the same *timeless* (*tim*) background, as *tim* alleles (*s-* and *ls-tim*) affect the overall level of reproductive arrest (S1 Table) [37]. We also overexpressed PDF in the same subset of cells (*Pdf*>*Pdf*), which again resulted in a significant decrease in the incidence of dormancy compared to controls (p<0.001; Fig 1A).

In addition to PDF, the s-LN_v_s co-express the neuropeptide sNPF. Since sNPF is widely present in the nervous system [53,55], we started by overexpressing this neuropeptide with a pan-neuronal driver. This manipulation (*elav>2xsNPF*) produced a very significant reduction in dormancy in the experimental flies compared to the controls (p<0.001; Fig 1B). Considering that both the *elav*-Gal4 and the UAS-*2xsNPF* lines carry the *ls-tim* allele (S1 Table), which is known to promote ovarian quiescence [37], the antagonistic effect of sNPF is quite dramatic. We then narrowed the overexpression of the neuropeptide specifically to the PDF^+^ neurons (*Pdf*>*2xsNPF*) and detected a similar and highly significant reduction of ovarian arrest in the experimental flies compared to the controls (p<0.001; Fig 1C). These results suggest that the Pdf-expressing tissues (the s-LN_v_s, l-LN_v_s, the PDF-Tri and the PDF-Ab) are making the major contribution to the inhibition of dormancy. To test the importance of the s-LN_v_s we used the *R6*-Gal4 driver [70] which is active in the s-LN_v_s and in some other neurons but not in the remaining PDF^+^ cells [71]. Again, *R6*>*2xsNPF* flies showed significantly reduced dormancy when compared to controls (p<0.001; Fig 1C). Thus, we conclude that sNPF, likely released from the s-LN_v_s, is involved in the negative regulation of gonadal arrest.

**Fig 2:**
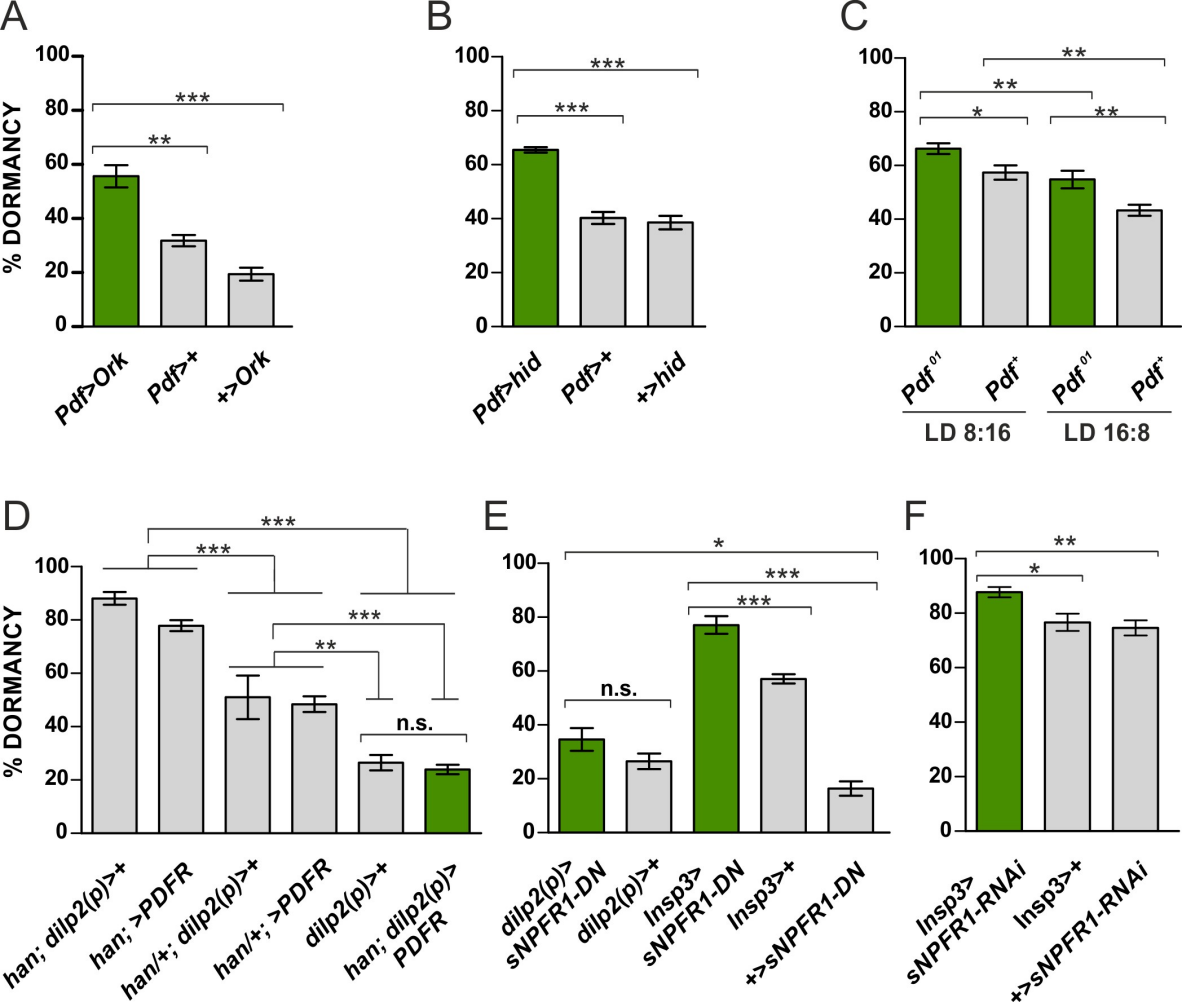
Inhibited neuronal activity of PDF^+^ neurons and impairment of sNPF signaling in the IPCs increase dormancylevels. (A) Expression of the potassium channel Ork (*Pdf*>*Ork*) enhances incidence of gonadal arrest in the experimental flies compared to control females. (B) Genetic ablation of PDF-expressing neurons (*Pdf*>*hid*) provokes a higher proportion of females entering dormancy compared to controls. (C) *Pdf*^*01*^ mutant females show enhanced ovarian quiescence levels compared to a congenic wild-type control at both summer and winter photoperiods (see text). (D) PDFR overexpression in IPCs in a *han/han* mutant background significant reduces dormancy compared to homozygous and heterozygous *han/+* controls, but not compared to the wild-type control *dilp2 (p)>+*, which also appears as one of the controls in Fig 2E. (E) Overexpression of a dominant negative form of sNPFR1 (UAS-*sNPFR1-DN*) in the IPCs under the control of an early and a later-expressed IPC driver (*dilp2(p)*-Gal4 and *Insp3*-Gal4, respectively) increases dormancy levels. (F) Downregulation of *sNPFR1* with RNAi using the *Insp3*-Gal4 driver significantly enhances reproductive arrest compared to controls. Numbers within bars indicate the number of dissected females considered in the dormancy assays. Data are presented as mean ± SEM. ANOVA on arcsine transformations, followed by *post-hoc* tests. ***p<0.001, **p<0.01, *p<0.05, n.s. not significant.

We then considered the opposite manipulation, namely reduced neuronal excitability, which ultimately results in reduced release of neuropeptides. Neuronal overexpression of the potassium channel Ork increases potassium efflux and causes membrane hyperpolarization, thereby preventing the firing of action potentials [72]. *Pdf*>*Ork* flies showed higher levels of ovarian arrest compared to controls (p<0.001; Fig 2A). Furthermore, genetically ablating the PDF^+^ neurons by overexpressing the pro-apoptotic protein hid (head involiution defective), *Pdf*>*hid*), also caused a larger proportion of females to undergo dormancy compared to controls (p<0.001; Fig 2B).

As the PDF neurons co-express more than just PDF we also examined whether the *Pdf*^*01*^ null mutation would alter dormancy levels at two photoperiods, LD8:16 and LD16:8. The experiment was performed on the *ls-tim* background. Fig 2C reveals that there is a significant genotype effect (p = 1.3 x 10^−4^) with *Pdf*^*01*^ mutants showing significantly elevated levels of ovarian quiescence plus a significant photoperiodic effect (p = 6 x 10^−6^), with no significant interaction, so the enhancement in dormancy occurs at both photoperiods equally. We also examined the effects of overexpressing the PDF receptor (PDFR) in the IPC cells using *dilp2(p)*-Gal4 [21,73] in a homozygous receptor mutant *han* background. This was compared to parental controls (*dilp2(p)*-Gal4 and UAS-*PDFR*) that were both homozygous and heterozygous for *han*, as well as the *dilp2(p)>+* wild-type control. All six genotypes were placed on the *s-tim* background. Fig 2D reveals that overexpression of *PDFR* causes a highly significant reduction of dormancy (p<0.001) compared to all the corresponding *han/han* and *han/+* controls, but is not significantly different from the *dilp2(p)>+* wild-type. Consequently, we appeared to have rescued the mutant phenotype in this genetic background. The heterozygous *han/+* background controls also show a highly significant reduction of dormancy compared to their corresponding homozygous mutant controls (both p<0.001) and the *dilp2(p)>+* wild type (p<0.01), further underscoring the dosage effect of PDFR on the phenotype. All of these experiments are consistent with the view that the s-LN_v_s modulate dormancy levels via the neuropeptides PDF and sNPF. However, since *Pdf*-Gal4 is also expressed in the non-circadian PDF-Tri and PDF-Ab neurons [39,41], an influence of the latter cannot be excluded.

### sNPFR1 signaling in the IPCs modulates reproductive arrest

The IPCs express sNPFR1 [54,57,68]; its activation by sNPF stimulates organismal growth by promoting the transcription of insulin-like peptides genes [54]. To investigate whether sNPFR1 signaling in the IPCs modulates ovarian arrest we expressed a dominant negative form of the receptor (UAS-*sNPFR1-DN*) under the control of two IPCs-specific drivers: *dilp2(p)*-Gal4 and *InsP3*-Gal4 [21,73]. The former drives gene expression from the 2^nd^ larval instar, the latter becomes active mainly after larval development [73].

Inhibition of sNFR1 from early larval stages (*dilp2(p*)>*sNPFR1-DN*) increased only marginally the proportion of dormancy (Fig 2E). However, when the receptor was repressed later in development (*InsP3*>*sNPFR1-DN*), a significantly higher proportion of flies showed gonadal arrest compared to controls (p<0.001; Fig 2E). Both drivers are specific for the 14 IPCs in the brain [21,73], so we speculate that different degrees of dormancy might reflect differences in the strength of the drivers or compensatory phenomena that occur early in development. We also used the *InsP3* driver to knockdown *sNPFR1*. We observed a significant increase in gonadal arrest in *InsP3> SNPFR1 RNAi* compared to the Gal4 (p = 0.017) and UAS controls (p = 0.006) (Fig 2F). These results are consistent with our previous observations regarding the antagonist nature of sNPF on this phenotype (Fig 1B and 1C), and also suggest a role for sNPF signaling in the IPCs in the regulation of reproductive arrest.

### No evidence for the involvement of the DLPs in the regulation of dormancy

The dorsal-lateral peptidergic neurons (DLPs) are 6–7 bilaterally symmetric neurons in the *Pars Lateralis* (PL) whose axons ends in the proximity of the IPCs [57]. The DLPs produce the neuropeptides Corazonin (Crz) and sNPF through which they modulate the activity of the IPCs, as the latter express the relevant receptors, CrzR (Corazonin Receptor) and sNPFR [57]. The DLPs affect survival, stress resistance and levels of circulating carbohydrates and lipids [57,74]. Since diapause is associated with marked changes of these parameters, we questioned whether the DLPs are involved in the regulation of this seasonal response. We used two DLP-specific *Crz*-Gal4 driver lines, *Crz*_*1*_-Gal4 and *Crz*_*2*_-Gal4, to overexpress Na^+^ChBac and sNPF, respectively. Both *Crz*_*1*_>*Na*^*+*^*ChBac* and *Crz*_*2*_>*2xsNPF* flies showed a reduction in the proportion of females undergoing gonadal dormancy (p<0.001) compared to one of the parental controls but not the other (S1 Fig). Therefore, although we cannot totally exclude an effect of the DLPs on ovarian arrest we can conclude that their involvement, if any, is not as robust as that observed for the PDF^+^ neurons.

### Terminals of the s-LN_v_s overlap with fine processes from the IPCs

**Fig 3:**
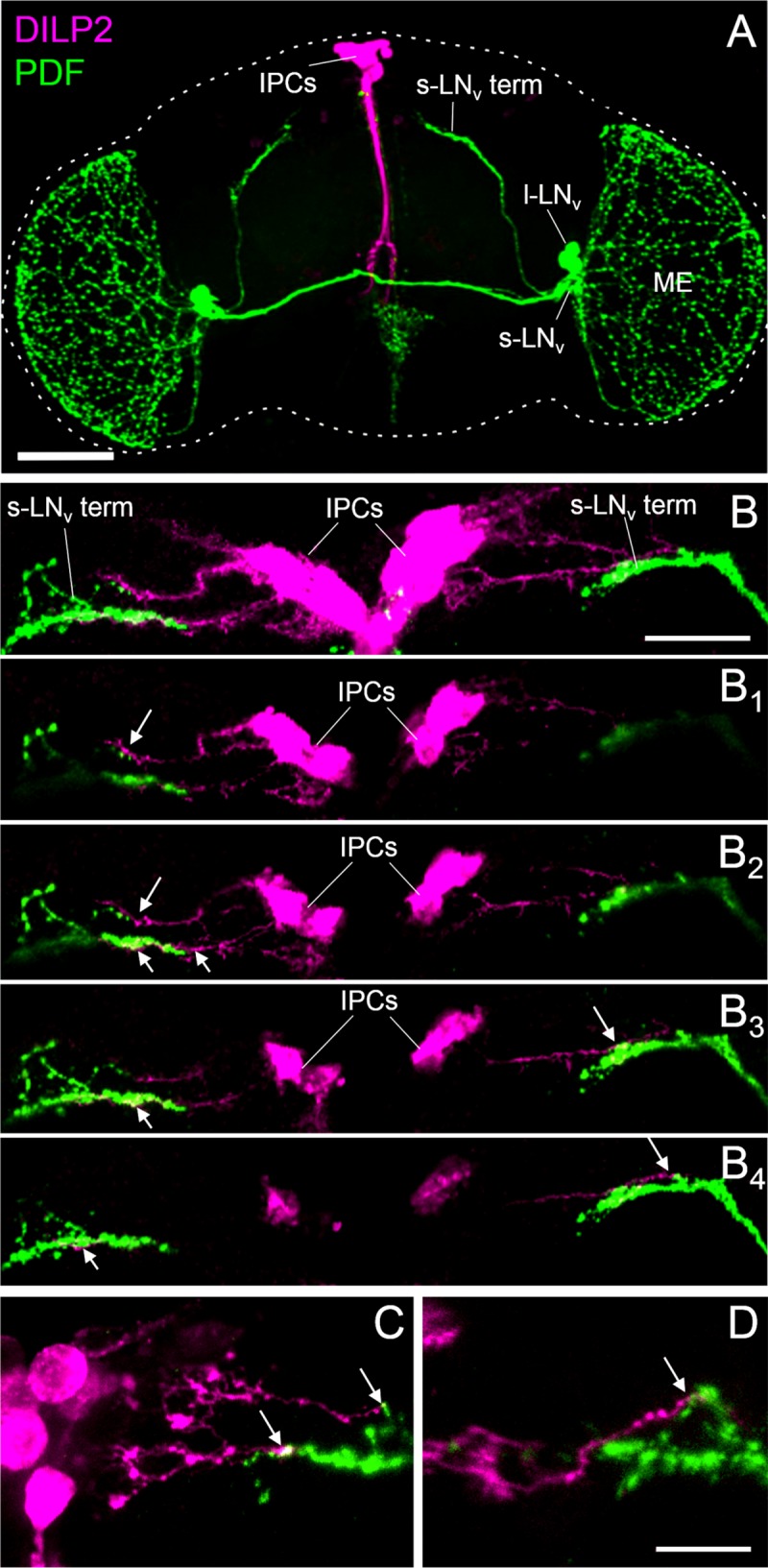
The terminals of the PDF positive s-LN_v_s overlap with dendrites of the insulin producing cells (IPCs). (A) Representative confocal image (overlay of Z-stacks through the entire brain) of a *Hu-S* fly brain double-stained with antibodies against PDF (green) and DILP2 (magenta). The brain is outlined by a dashed line. Me = medulla. Scale bar = 100 μm. (B) Representative confocal image of the dorsal part of *dilp2(p)>GFP* brains double stained with antibodies against PDF (green) and GFP (magenta) (overlay of 10 Z-stacks). Processes of the insulin producing cells (IPCs) overlap with the terminals of the small ventrolateral neurons (s-LN_v_ term) as can be seen in single confocal stacks of 2 μm thickness (B_1_-B_4_). Every second single stack of the posterior part of (B) is shown from anterior to posterior. White arrows indicate close proximity between IPCs and s-LN_v_ terminals. Scale bar = 20 μm. (C-D) Labeling of the IPCs with the dendritic marker DenMark [75] (*dilp2(p)>DenMark*) indicate that the IPC fibers contacting the s-LN_v_ terminals are of dendritic origin. Representative confocal images of two different brains are shown. Scale bar 10 μm.

Next, we asked whether the dorsal terminals of the s-LN_v_s in the dorsal vicinity might be close enough to the IPCs to enable such sNPF and PDF signaling. By performing ICC with anti-DILP2 and anti-PDF we could not see direct contacts overlapping between the s-LN_v_s and the IPCs (Fig 3A). However, when we expressed GFP in the IPCs (*dilp2(p)>GFP*), we observed that the dorsal projections of the s-LN_v_s are in close proximity to fine processes originating from the IPCs (Fig 3B). To test whether these fine processes are dendrites, we expressed the dendritic marker DenMark in the IPCs (*dilp2(p)>DenMark*) [75]. We found prominent labeling in the IPC processes, indicating that they are of dendritic origin (Fig 3C and 3D). This explains why we could not see them by anti-DILP2 labeling.

### IPCs respond to bath-applied PDF and sNPF

The IPCs are neurosecretory cells that are crucial for initiating seasonal responses [13,17,19,20]. Since we have shown that PDF and sNPF have a modulatory effect on diapause (Fig 1), and that PDF^+^ and sNPF^+^ neurons appear to contact the IPCs (Fig 3B)[57], we asked whether the IPCs can respond directly to these neuropeptides. The PDF receptor signals primarily via cAMP [59,61,76], whereas the signaling cascade following activation of the receptor for sNPF uses cAMP, at least in part [77–79]. Thus, we expressed a genetically encoded fluorescence resonance energy transfer (FRET) based cAMP sensor in the IPCs (*dilp2(p)*>*Epac1camps*) to monitor real-time cAMP levels [62]. A similar experimental design had previously been adopted with success to investigate the presence of the PDF receptor in clock neurons [62].

**Fig 4:**
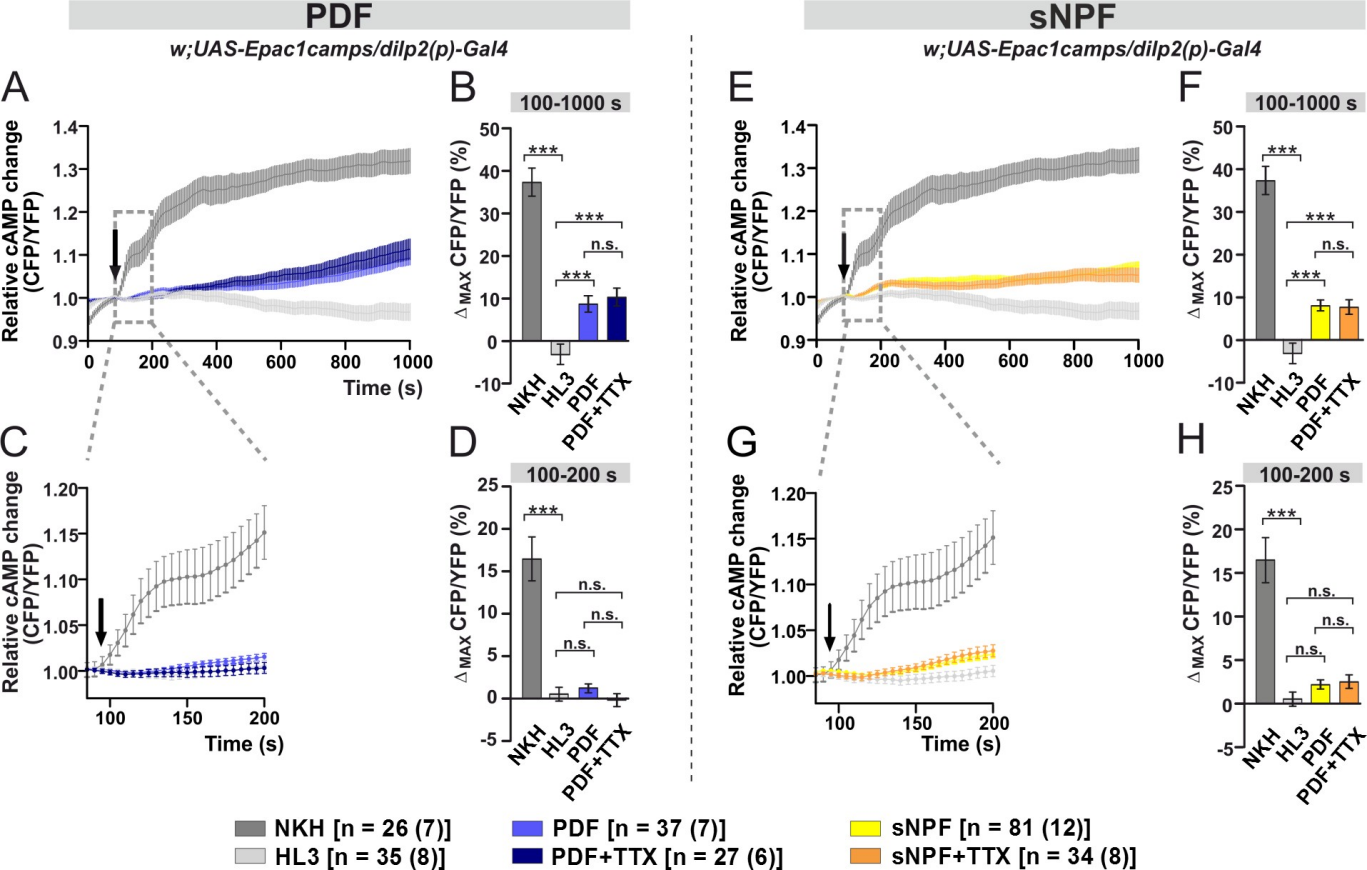
Bath-applied PDF and sNPF induce cAMP increases in the IPCs. Live optical imaging in flies expressing a cAMP sensor in their IPCs (*dilp2(p)*>*Epac1camps*). Average inverse FRET traces (CFP/YFP) in IPCs reflecting intracellular changes in cAMP levels. Substances were bath-applied to freshly dissected fly brains at 100 s (indicated by black arrow). Application of 10^−5^ M adenylate cyclase activator NKH477 (NKH, dark gray) induced a robust increase in cAMP, indicating that the general procedure was working. As a negative control, hemolymph-like saline (HL3) was applied. (A) Bath-applied PDF (10^−5^ M) evokes cAMP increases in the IPCs (light blue), suggesting a possible functional connection between PDF^+^ cells and IPCs. Similar increase was observed when PDF was applied in the presence of 2**μ**M sodium channel blocker tetrodotoxin (TTX; dark blue), indicating a direct connection. (B) Maximum inverse FRET changes quantified for each individual neuron and averaged for each pharmacological treatment between 100–1000 s. (C) A close-up of the immediate changes in cAMP levels occurring from the application point until 200 s. No significant changes can be observed when PDF or PDF+TTX were applied. (D) Maximum inverse FRET changes from 100–200 s. (E) Bath-applied sNPF (10^−5^ M) generates cAMP rises in the IPCs (yellow). Similar increase occurs in the presence of TTX (orange), thus suggesting a direct connection. (F) Maximum inverse FRET changes between 100–1000 s. (G) Magnification of the immediate changes between 100–200 s. (H) Maximum inverse FRET changes between 100–200 s. The legend shows the color code of the different treatments and the number of neurons in the dissected brains (in parentheses) considered in this analysis. Data are shown as mean ± SEM. Kruskal-Wallis test followed by Bonferroni-corrected Wilcoxon pairwise-comparisons. ***p<0.001, **p<0.01, *p<0.05, n.s. not significant.

We bath-applied 10 **μ**M of synthetic PDF to acutely dissected fly brains. This resulted in a slow rise in the intracellular amount of cAMP, measured as the average FRET signal between 100 and 1000 s after the application of the neuropeptide (light-blue curve and bar; Fig 4A and 4B). cAMP FRET signals were 10% *ca*. higher (100–1000 s; p<0.001) compared to the negative (modified minimal hemolymph-like solution, HL3, light-grey curve and bar; Fig 4A and 4B) control. A similar increment was also observed after presenting PDF together with the sodium channel blocker tetrodotoxin (TTX, dark-blue curve and bar, 100–1000 s; p<0.001; Fig 4A and 4B). The latter prevents neuronal communication, suggesting that PDF activates the IPCs directly. However, the short-term (100-200s) response to PDF was unchanged compared to the negative control, either with or without TTX (Fig 4C and 4D).

Similar observations were made upon the application of 10 **μ**M synthetic sNPF. This resulted in a slow but significant cAMP increase in the IPCs either in the absence (yellow curve and bar, 100–1000 s; p<0.001) or in the presence (orange curve and bar, 100–1000 s; p<0.001) of TTX, reflecting a direct activation of the IPCs by sNPF (Fig 4E and 4F). Moreover, as we saw for PDF, the short-term responses to sNPF did not differ from the negative control (Fig 4G and 4H).

**Fig 5:**
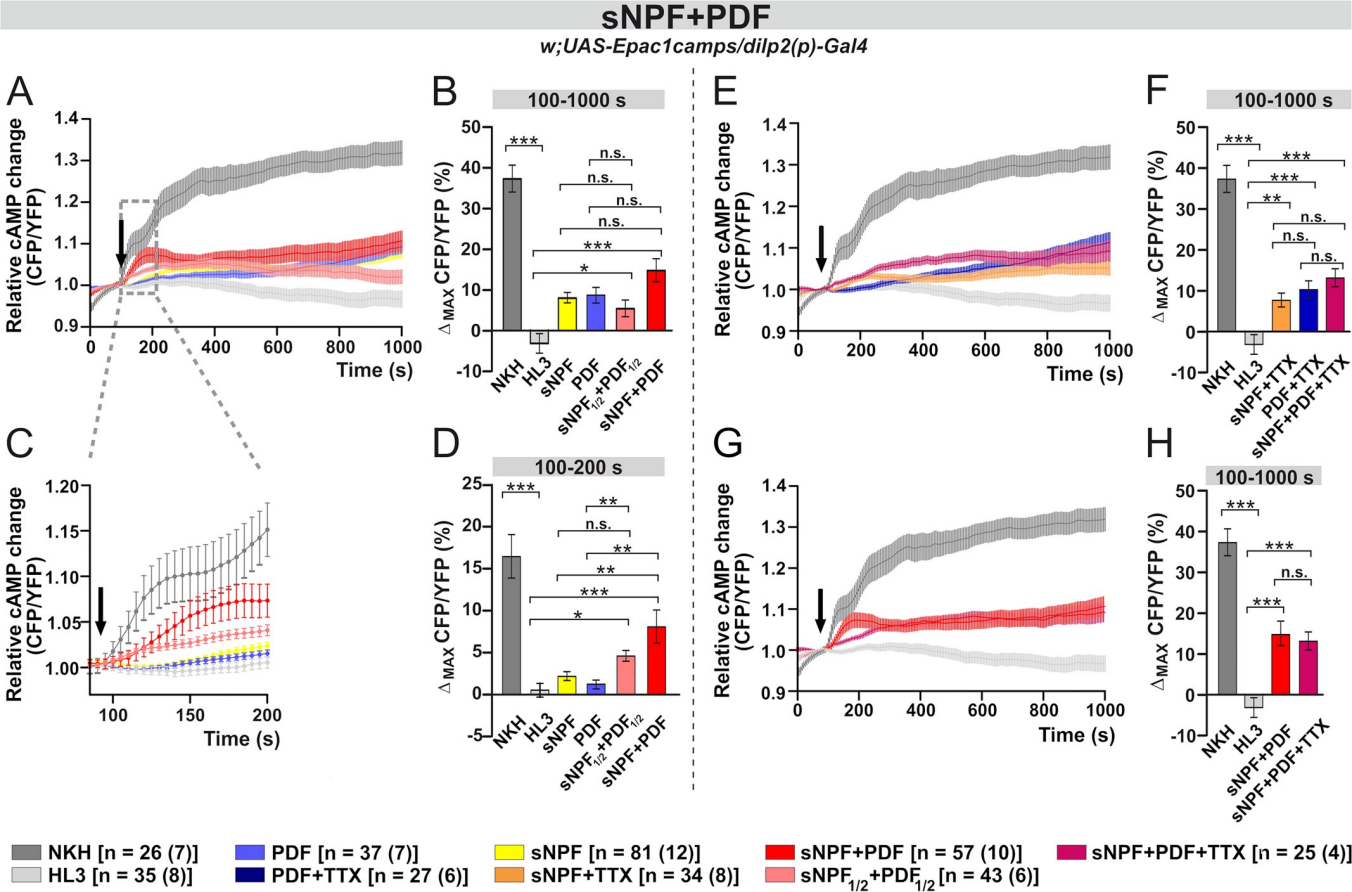
The co-application of PDF and sNPF increase the cAMP response. (A) Co-application of sNPF and PDF evokes large cAMP increases in the IPCs (red), which remain significant even when concentrations of peptides are halved (rose). (B) Maximum inverse FRET between 100–1000 s. (C, D) Immediate cAMP responses (100–200 s) reveal a large, rapid increase due to the sNPF+PDF co-application. (E) Co-application of sNPF and PDF induces cAMP increases in the IPCs also in the presence of TTX (magenta), and this response seems to be larger in the time interval of 200–600 s than the effect triggered by the application of the single peptides with TTX. (F) Maximum inverse FRET changes from 100–1000 s. (G) The effect of the sNPF+PDF co-application seems to be, at least partially, due to a direct activation of the IPCs. The initial increase is apparently reduced when TTX is present (magenta). (H) Maximum inverse FRET changes from 100–1000 s. The legend shows the color code of the different treatments and the number of neurons in the dissected brains (in parentheses) considered in this analysis. Data are shown as mean ± SEM. Labeling as in Fig 4.

However, when we applied sNPF and PDF together (sNPF+PDF), at a concentration of 10 **μ**M for each peptide, we recorded an increase in cAMP FRET signal, reaching a level ~15% higher than that for the negative control (red, 100–1000 s; p<0.001; Fig 5A and 5B). Moreover, the short-term response was particularly distinctive compared to the applications of single peptides, revealing a ~8% increase in cAMP FRET signals (red curve and bar; 100–200 s; p<0.01; Fig 5C and 5D). We repeated the experiment but halving the concentration of each peptide to 5 **μ**M (sNPF_1/2_+PDF_1/2_). This also resulted in a significant increase in cAMP (pink curve and bar, 100–1000 s; p<0.05; Fig 5A and 5B). However, we noticed that following an initial increase, after 400 s the concentration of cAMP slowly declined (pink curve and bar; Fig 5A). Focusing on the short-term response, the co-application of (sNPF_1/2_+PDF_1/2_) resulted in higher cAMP FRET signals compared to the bath treatment with single peptides; however, the difference was statistically significant only with regard to PDF (100-200s; p<0.01; Fig 5C and 5D).

We also tested (sNPF+PDF+TTX) and found an increase of ~13% in the amount of cAMP FRET signal compared to the negative control (magenta curve and bar, 100–1000 s; p<0.001; Fig 5E and 5F). This response was not statistically different from single peptide applications in the presence of TTX although in the interval 300–600 s the levels of cAMP for (sNPF+PDF+TTX) were higher (Fig 5E and 5F). Interestingly, although there were no differences in cAMP levels when comparing the application of sNPF+PDF with or without TTX in the interval 100–1000 s, the distinctive short term response disappeared in the presence of TTX (Fig 5G and 5H). This suggests that the short-term response of the IPCs to the combined application of (sNPF+PDF) is indirectly mediated by other cells.

**Fig 6:**
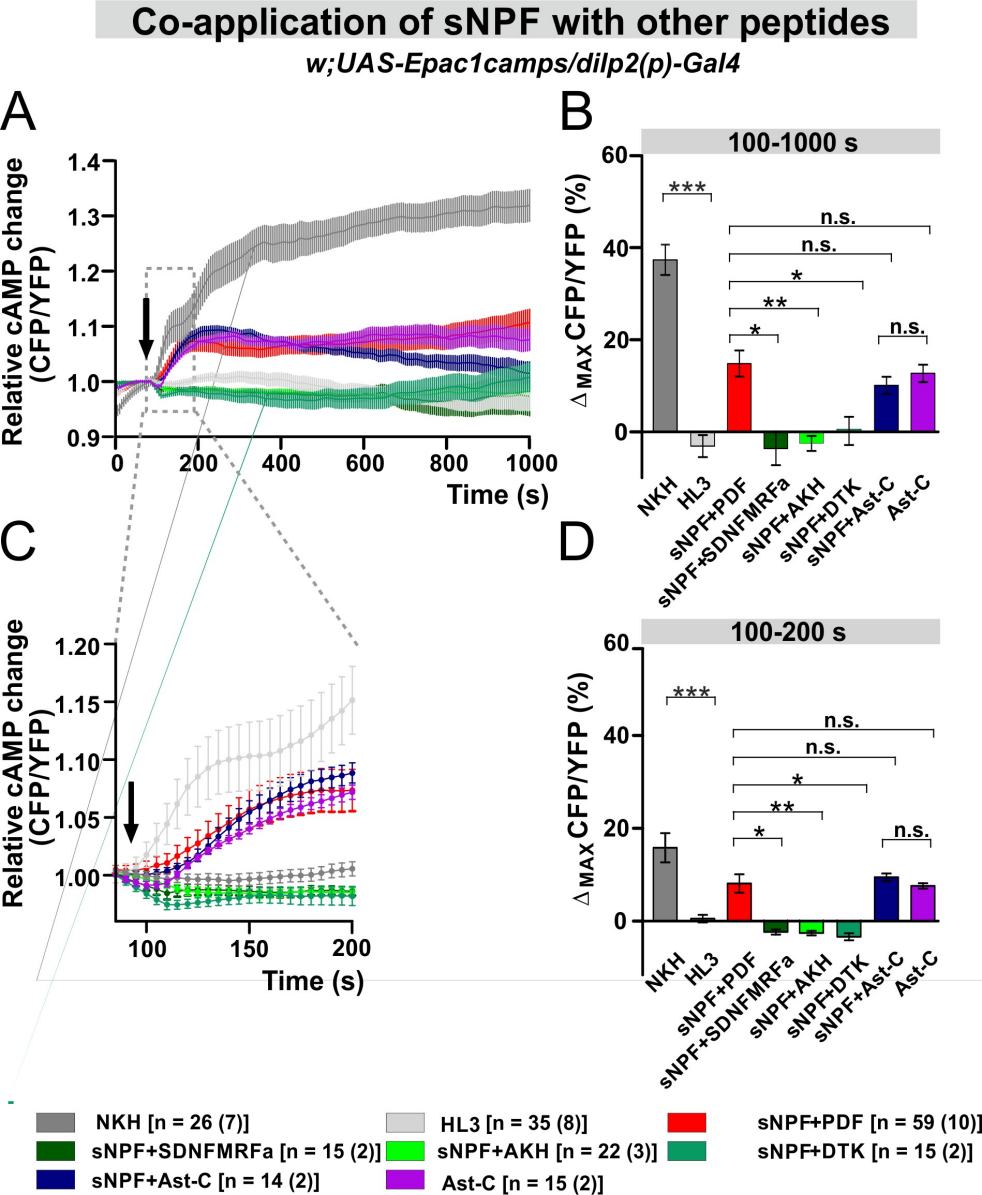
Co-application of sNPF with other *Drosophila* peptides suggests that sNPF and PDF may have a unique interaction. (A) Average inverse FRET traces (CFP/YFP) of IPCs reflecting intracellular cAMP changes. 10^−5^ M synthetic sNPF was co-applied with PDF, SDNFMRFa, AKH (adipokinetic hormone), DTK (Drosophila tachykinin), Ast-C (Allatostatin-C) /10^-5^M for each/ or 10^−5^ M Ast-C was added alone. The black arrow indicates the application point of the different substance. NKH: adenylate cyclase activator, used as positive control. HL3: hemolymph-like saline, used as negative control. (HL3) (B) Maximum inverse FRET changes quantified for each individual neuron and averaged for each treatment from 100 s until 1000 s. Statistical comparison revealed that co-application of sNPF with SDNFMRFa, AKH, and DTK resulted in a significant decrease of cAMP levels compared to the sNPF+PDF co-application. sNPF+Ast-C led to a significant increase in the level of cAMP, however similar change was observed in the case of Ast-C application alone. (C) A close up of the immediate cAMP level changes occurring from the application point until 200 s. (D) Maximum inverse FRET from 100 s until 200 s. The legend indicates the color code of the different treatments and the number of neurons in the dissected brains (in parentheses) considered in this analysis. ***p<0.001, **p<0.01, *p<0.05, n.s. not significant.

We assessed whether the larger increase in cAMP levels induced in the IPCs by the co-application of sNPF+PDF is a specific response. We co-applied sNPF with the following neuropeptides: SDNFMRFamide (SDNFMRFa), adipokinetic hormone (AKH), *Drosophila* tachykinin (DTK) and allatostatin-C (Ast-C). AKH and DTK were chosen because they are known regulators of IPC activity [21,73,80,81]. When sNPF was co-applied with SDNFMRFa, AKH or DTK, the levels of cAMP were significantly reduced compared to the sNPF+PDF co-application (p<0.05, p<0.01, p<0.05, respectively) and were indistinguishable from the negative control (Fig 6). Conversely, the co-application of sNPF+Ast-C resulted in a significant increase in the amount of cAMP but the addition of Ast-C alone was sufficient to evoke similar cAMP responses in the IPCs (Fig 6). To our knowledge, no data are available on the distribution of the Ast-C receptor in *Drosophila*, and no link between IPCs and Ast-C has been reported previously. In summary, our data show that the strong and rapid responses of the IPCs we observed are unique to the co-application of PDF and sNPF. In particular, they are not caused either by the combination of other peptides or by receptor cross-activation due to high peptide doses applied.

**Fig 7:**
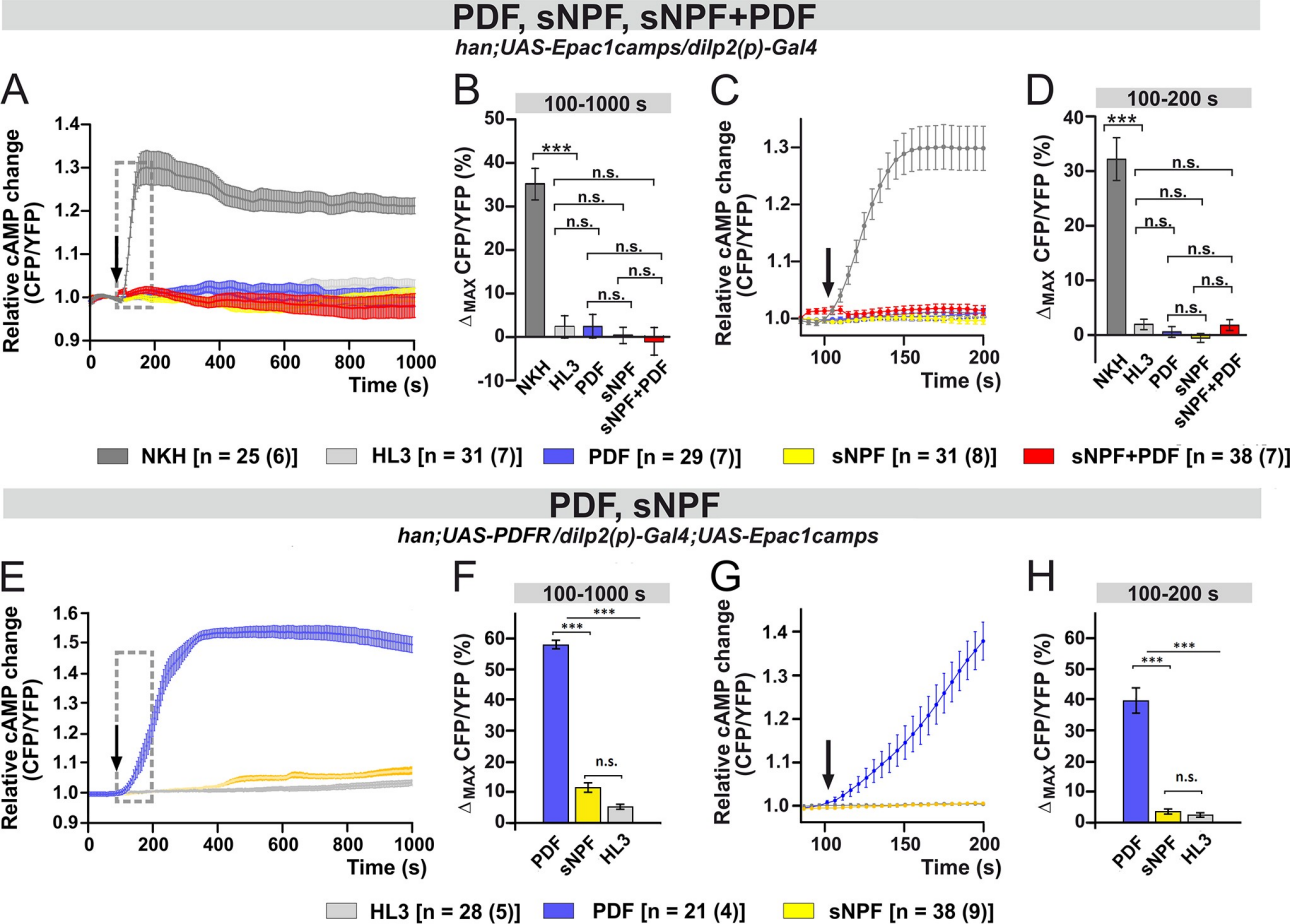
The responses of the IPCs to PDF and sNPF are absent in the PDF receptor mutant *han*, but only the response to PDF is mediated by the PDF receptor. (A-D) Live imaging in *han* mutants expressing a cAMP sensor in their IPCs (*han; dilp2(p)*>*Epac1camps*). In the mutant background, the effects of both neuropeptides are abolished. (A-B) Maximum inverse FRET changes from 100–1000 s. Short-term responses are all abrogated in *han* flies. (C-D) Maximum inverse FRET changes from 100–200 s. (E-H) Live imaging in *han* mutants with the PDF receptor rescued in the IPCs. The expression of *han* in the IPCs induces very strong responses to PDF but not to sNPF (E-F) Maximum inverse FRET changes from 100–1000 s. (G-H) Maximum inverse FRET changes from 100–200 s. Labeling as in Fig 4.

To verify whether the responses to PDF were mediated by its receptor, we carried out the treatment in the PDFR-null (*han*) [59] background (*han; dilp2(p)*>*Epac1camps*). The application of PDF in the mutant no longer evoked an increase of cAMP (light-blue; 100–1000 s; Fig 7A–7D), and surprisingly, neither did sNPF (yellow curve and bar; 100–1000 s; Fig 7A–7D). The rapid increase in cAMP levels induced by the co-application of sNPF+PDF was also obliterated in the mutant (red curve and bar; 100–200 s; Fig 7C and 7D). To test whether sNPF acts via PDFR, we rescued PDFR expression in the IPCs in the *han* mutant background (*han; dilp2(p)*>*PDFR; Epac1camps*). In these flies, IPCs strongly responded to PDF, but not to sNPF. It is therefore unlikely that sNPF signals via PDFR (Fig 7E–7H). We observed a slight increase in cAMP ~400s after sNPF application, but this was not significantly different from the negative controls (yellow curve and bar; 100–1000 s; Fig 7E and 7F). The fast response to sNPF was completely absent (yellow curve and bar; 100–200 s; Fig 7G and 7H). This shows that the absence of the response to sNPF in the *han* mutant cannot be directly attributed to the loss of PDFR.

### sNPF but not PDF increases Ca^2+^ levels in the IPCs

**Fig 8:**
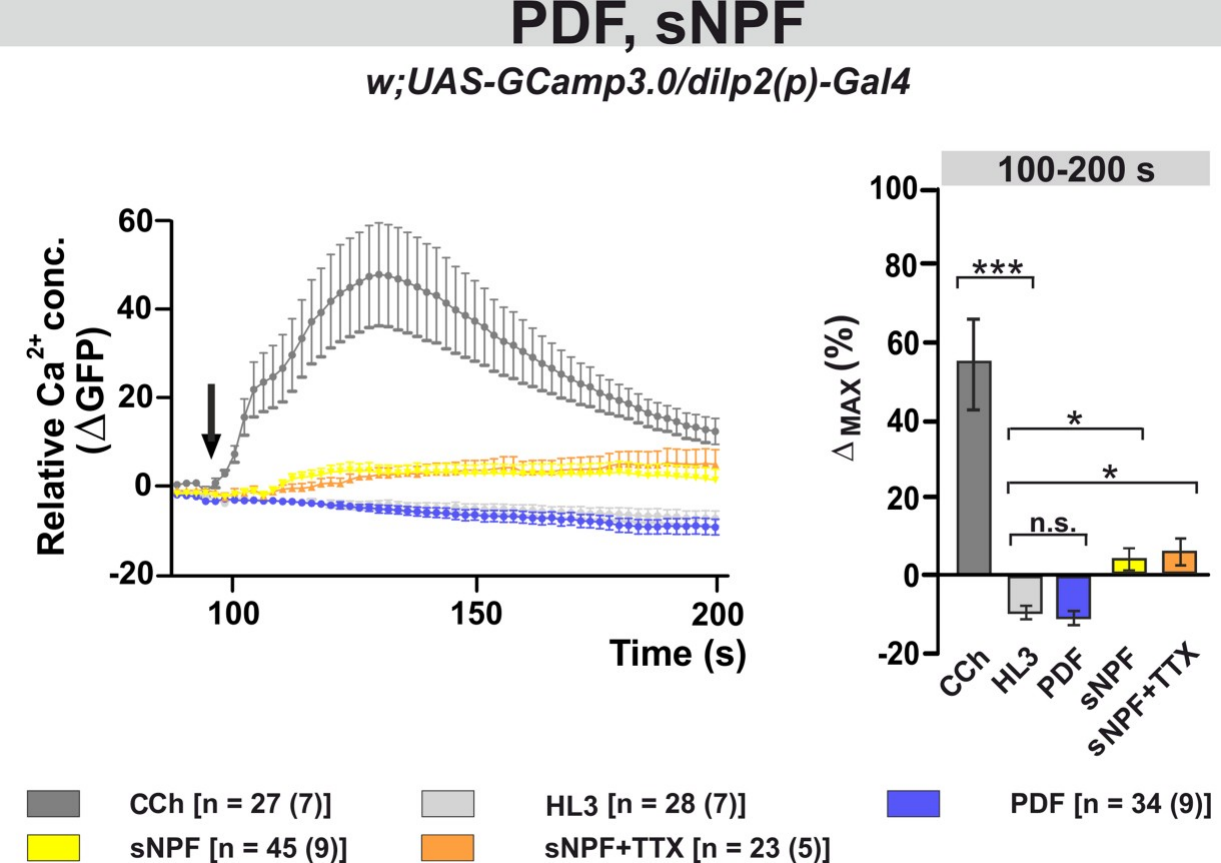
The neuropeptide sNPF induces a small increase in the intracellular Ca^2+^ level, while PDF has no effect. Left panel: Average changes in GFP fluorescence of IPCs reflecting intracellular changes in Ca^2+^ levels. Substances were bath-applied to freshly dissected fly brains at ~100 s (indicated by a black arrow). The cholinergic agonist carbamylcholine (1 mM CCh) was used as positive control, which induced a robust increase in Ca^2+^, indicating that the general procedure was working. As a negative control, hemolymph-like saline (HL3) was applied. Application of 10–5 M synthetic PDF peptide did not alter Ca^2+^ levels, while 10–5 M sNPF induced a small but significant increase in Ca^2+^ levels. This calcium response seems to be due to direct activation of the IPCs, since it was not blocked in the presence of the sodium channel blocker TTX (2**μ**M). Right panel: Maximum Ca^2+^ changes (%) for each individual neuron were calculated and averaged for each pharmacological treatment from 100 s until 200 s. Statistical comparison revealed significant increases in Ca^2+^ levels compared to the negative control (HL3) for CCh, sNPF and sNPF+TTX, while PDF had no effect. Data are presented as mean ± SEM. The color code of the different treatments and the number of neurons [n] and dissected brains (n) considered in this analysis are shown below the panels. Kruskal-Wallis test followed by Bonferroni-corrected Wilcoxon pairwise-comparisons. ***p<0.001, *p<0.05, n.s. not significant.

In several cell types PDF signals primarily through cAMP rather than calcium [59,61,76]. Here we tested whether PDF or sNPF affect the level of intracellular Ca^2+^ in the IPCs by expressing the genetically encoded Ca^2+^ sensor GCaMP3.0 (*dilp2(p)*>*GCaMP3*.*0*) [82] Incubating PDF with freshly dissected brains did not produce change in Ca^2+^ levels, which were indistinguishable from the negative control (Fig 8). However, the bath application of sNPF induced a small but significant increase in the Ca^2+^ signal (100-200s, 3.4 ± 2.8%, p<0.05). This was still detectable in the presence of TTX (100-200s, 5.4 ± 3.5%, p<0.05), suggesting that this response is not mediated by interneurons but is due to the direct activation of the IPCs by sNPF (Fig 8).

## Discussion

### Genetic manipulations of PDF-expressing neurons modulate dormancy

We have constitutively activated the PDF^+^ neurons with Na^+^ChBac [69] and observed a significant reduction in levels of gonadal arrest (Fig 1A). Since this treatment increases membrane excitability, it likely increases release of both PDF and sNPF, whose overexpression also led to a significant reduction in dormancy (Fig 1B and 1C). Manipulations with opposite effect, namely reduction of membrane excitability of the PDF^+^ neurons through overexpression of the K^+^ channel Ork [72] or induction of cell death by overexpression of the pro-apoptotic gene *hid*, resulted in an enhanced dormancy response (Fig 2A and 2B). Furthermore, the *Pdf*^*0*^ mutant showed a significantly elevated response compared to controls and overexpressing *PDFR* in the IPCs significantly reduced gonadal arrest on both heterozygous and homozygous mutant *han* backgrounds to wild-type levels. These results consistently support a model where PDF and SNF act to antagonise dormancy, possibly by enhancing dILP expression. When we expressed a dominant negative form of the sNPF receptor in the IPCs, or downregulated the receptor with RNAi, higher dormancy induction was observed especially when using a driver that is active later in development (Fig 2E and 2F). Numerous sNPF-expressing neurons can potentially target the IPCs, including the PDF^+^ sLN_v_s and the DLPs. However, the manipulations of the DLPs did not affect reproductive quiescence (S1 Fig).

One puzzling aspect of the results is that they appear, at least superficially, to contradict a recent study in which *Pdf*^*01*^ females showed relatively low levels of dormancy and did not reveal a photoperiodic effect [83]. The LD cycles used in these experiment were LD16:8 versus LD10:14 so not as extreme as ours and this might have had a damping effect on any photoperiodicity. Furthermore, the low dormancy levels of the mutant females suggested that the mutation might have been on the *s-tim* background. A congenic wild-type was not compared to the mutant, so it is difficult to predict whether such a control would have had lower levels of reproductive arrest which would be consistent with our findings. In addition, the further stress of starvation was incorporated into the experimental paradigm. It would therefore be of interest to examine whether our results, which suggest that the neuropeptides released by clock cells antagonise reproductive quiescence, can be generalized under a variety of different unfavourable environmental conditions.

### The IPCs respond to PDF and sNPF

Using a whole brain *ex-vivo* preparation, we observed that the IPCs responded to bath-applications of sNPF and PDF with increasing cAMP levels. The responses persisted when synaptic connections with the rest of the brain were inhibited by TTX, suggesting a direct effect on the IPCs of these neuropeptides (Fig 4A–4H). Expression of sNPFR1 in the IPCs had already been described [53,54,57,68]. Interestingly, sNPFR1 couple to more than one Gα-protein subtype, since both excitatory (through G_s_α [77,78]) and inhibitory effects (through G_o_α [79]) on cAMP levels have been documented. In addition, sNPF can signal by suppressing Ca^2+^ in some circadian clock clusters [42] and peptidergic PTTH neurons [84]. Here, we found that sNPF increased Ca^2+^ levels in the IPCs showing that it can also have activating Ca^2+^ effects. Similar multiple G-protein coupling has also been reported for the neurokinin-1 and -2 receptors [85,86], as well as for the glucagon receptor in human atrial membranes [87].

The expression of the PDFR in the IPCs is less well-characterized. A previous study using fluorescent *in situ* hybridization reported prominent *PDFR* expression in the PI. However, it did not identify the *PDFR* positive cells [60]. Moreover, another study suggested that the cAMP responses evoked in the IPCs by PDF are not as robust as those registered in clock neurons [62]. Possibly, lower ligand efficacy might simply reflect less PDFR in these cells. The strong cAMP responses we observed after driving PDFR in *han* mutants under control of *dilp2(p)*-Gal4 supports this conclusion (Fig 6E–6H). On the other hand, there are 14 IPCs in the PI which show heterogeneous protein and neuropeptide composition [29,88], and rhythmic electrophysiological parameters [33]. Thus, it is possible that, like the s-LN_v_s [89], the responsiveness of the IPCs to PDF is also influenced by time of day. In any case, here we found significant cAMP increasing effects of PDF on the IPCs.

When we applied sNPF and PDF together, the short-term (100–200 s) response of the IPCs to the combined peptides was greater than the sum of their separate activities, pointing to a synergistic effect between the two molecules (Fig 5A–5D). Similar interactions were not observed when sNPF was co-applied with other *Drosophila* neuropeptides, suggesting that the interaction of sNPF and PDF is specific. Nevertheless, the addition of TTX dampened the short-term response, suggesting that additional cells participate in the synergism between sNPF and PDF (Fig 5E–5H). Interestingly, *han* mutants lacking PDFR did not respond to PDF, sNPF or sNPF+PDF co-application (Fig 7A–7D). For sNPF, these results are puzzling, but since the rescue of PDFR in the IPCs only restored the response to PDF but not to sNPF, we conclude that sNPF does not act via PDFR. Still, we cannot completely exclude a cross-talk between PDFR and sNPFR1. For instance, there is evidence that GPCRs can engage in homo- or hetero-oligomeric complexes, resulting in cooperativity (reviewed in [90]). Alternatively, an interaction downstream of the receptors may occur, for example at the level of the signalosomes. PDFR and sNPFR associate with specific and different signalosomes that may have enhancing or opposing effects on cAMP levels [76–79,91]. However, further studies are required to investigate these possibilities using experimental settings that are closer to physiological conditions than bath applications of peptides. Interestingly, sNPF (produced in the s-LN_v_s and in a subset of dorsal lateral neurons) and PDF interact on clock neurons and set the phase of their cytosolic Ca^2+^ rhythms according to neuron cluster [42]. PDF primarily regulates Ca^2+^ rhythms in the LN_d_ and DN3 clusters, while sNPF orchestrates that of the DN1 [42]. Thus, the two signaling pathways act dynamically to facilitate the right timing of circadian neuronal activities. Similar complex regulation could also influence the seasonal clock system.

While the *Pdf* mRNA and protein do not cycle, it has been reported that PDF cycles at the nerve terminals with a circadian rhythm [39]. Thus, we presume that the daily rhythmic stimulation of the IPCs under normal summer conditions of warm days and long photoperiods maintains the expression of dILPs and suppresses dormancy. A simple model would have low temperatures and short photoperiods reducing the expression of PDF and sNPF from the sLNvs terminals, which might be expected to reduce IPC activation and enhance reproductive arrest levels. This could mean that the transcription/translation of these neuropeptides could be reduced under colder conditions, or alternatively, that their release was reduced from the sLNv terminal. This scenario might imply higher levels of PDF within the sLNv soma if it is sequestered there. Alternatively, the receptors for PDF and SNPF on the IPCs might be less sensitive to their ligands at lower temperatures, which would have the same effect on reproductive arrest. Future studies are required to examine the dynamic nature of PDF/SNPF and receptor expression under simulated winter conditions.

PDF and sNPF–most likely from the s-LN_v_s can maintain *D*. *melanogaster*, originally a tropical species, in the reproductive state. However, it is intriguing that high-latitude *Drosophila* species such as *D*. *montana*, *D*. *littoralis*, *D*. *ezoana* and *D*. *virilis* lack PDF in the s-LN_v_s [92–95]. These species have a high incidence of reproductive arrest even under long-daylengths, an adaptation to the low temperatures even under summer photoperiods at these clines. For example, *D*. *ezoana* enters diapause when day-length falls below 16 hours [96]. Although we do not know whether the s-LN_v_s of the high-latitude species still express sNPF, we speculate that the lack of PDF-signaling to the IPCs of these species might facilitate the termination of the reproductive state under short-day condition, and induce ovarian arrest [97]. The role of the s-LN_v_s as a source of PDF and sNPF may thus provide the entry point into the neuronal mechanism that allows *D*. *melanogaster* to detect the environmental conditions that predispose them to reproductive dormancy.

## Materials and methods

### Fly stock and Maintenance

Flies were reared at 23°C, 70% relative humidity, under 12-hour light/12-hour dark cycles (LD 12:12) on cornmeal standard food. The following, previously described fly strains were used: *Hu-S* Dutch natural population [37], *han*^*5304*^ [59], UAS-*PDFR* [59], *gal1118 Gal4* enhancer trap [98], *R6*-Gal4 [70], UAS-*Epac1camps* [62], UAS-*hid* [99], UAS-*Pdf* [41], and *UAS-DenMark* [75]. *InsP3*-Gal4 was a gift from Michael J. Pankratz [73], *dilp2(p)*-Gal4 (*p*, *precocious*) was a gift from Eric J. Rulifson [21], UAS-*2xsNPF* [53] and UAS-*sNPFR1-DN* [54] were kindly provided Kweon Yu. Out of these lines, we newly combined *han*^*5304*^; *dilp2(p)*-Gal4, *han*^*5304*^;;UAS-*Epac1camps*, and *han*^*5304*^;UAS-*Epac1camps/CyO*;UAS-*PDFR*. The transgenic line *Pdf*-LexA,LexAop-*CD4*::*GFP*_*11*_/*CyO*;UAS-*CD4*::*GFP*_*1-10*_/*TM6b* for GRASP was a gift from François Rouyer. UAS-*OrkΔ-C* (designated as UAS-*Ork* [72]) and UAS-*Na*^*+*^*ChBac* [69]) were gifts from Michael B. O’Connor. *Pdf*^*01*^ mutants were backcrossed for 8 generations to a natural wild-type *ls-tim* line from The Netherlands [37]. The following lines were ordered from Bloomington Drosophila Stock Center: *Canton-S* wild-type strain (*#1*), *Crz*_*1*_-Gal4 *(#51976*), *Crz*_*2*_-Gal4 *(#51977*), *Elav*^*C155*^-Gal4 (designated as *elav*-Gal4, *#458*), *Pdf*-Gal4 (*#6900*), UAS-*CD8-GFP* (*BDSC #5137*), and *Oregon-R* (*#2376*; used as control for experiments with *han* mutant).

In order to use a control in which the specific transgene (Gal4 or UAS) was in the same condition of heterozygosis as in the experimental line, we have crossed all the parental lines to the *w*^*1118*^ strain (generic genotype *w*^*1118*^*;P-element/+*). In *Drosophila*, two allelic variants of the *timeless* circadian clock gene (*s-tim*/*ls-tim*) were found to significantly influence reproductive arrest: *ls-tim* allele promotes reproductive quiescence at every photoperiod [37,38]. Considering this modulatory effect, controls were generated according to the *tim* allele of the UAS and Gal4 strains used in the experiments (see below for PCR *timeless* genotyping), with the help of *w*^*1118*^*; s-tim* (gift from Matthias Schlichting) and *w*^*1118*^; *ls-tim* (from our lab) lines that express either the *s-* or *ls-tim* isoform.

### Dormancy assay

All fly stocks and crosses used for dormancy assays were maintained at 23°C in LD 12:12. Newly eclosed flies (within 5 h post-eclosion) were placed in plastic vials, and immediately subjected to 12°C in LD 8:16. After 11 days, flies were killed in abs EtOH, and ovaries of females were dissected in PBS. Dormancy levels were scored by considering the absence of yolk deposition in the ovarian follicles [7]. These data were presented as the proportion of females with ovarian arrest among all the dissected individuals. On average, 5 replicates of n>60 flies were dissected for each genotypes.

### Immunocytochemistry

Flies were reared at 23°C in LD 12:12, and newly eclosed individuals were placed in short days (LD 8:16) at different temperatures (12, 18, 23°C) for 11 days. Females were collected at ZT1 (*Zeitgeber* Time 1, 1 h after light-on), and immediately fixed in 4% paraformaldehyde (PFA) in PBS for 100 min at room temperature (RT). After 3 washes in PBS, brains were dissected in ice-cold PBS, fixed in PFA 4% for 40 min at RT, and subsequently washed 6 times in PBS containing 0.3% Triton X-100 (PBS-T). Next, samples were permeabilized in 1% PBS-T, followed by an overnight blocking step in 1% bovine serum albumin (BSA) in 0.3% PBS-T at 4°C. Afterwards, brains were incubated in primary antibody solution (diluted in 0.1% BSA, 0.3% PBS-T) for 3 days at 4°C. After 6 washes in 1% BSA in 0.3% PBS-T at RT, another blocking step was performed in 1% BSA in 0.3% PBS-T at 4°C, followed by hybridization with the secondary antibody (diluted in 0.1% BSA, 0.3% PBS-T) overnight at 4°C. The primary antibodies used in this study: mouse anti-GFP (1:500; Thermo Fisher Scientific), rabbit-anti-GFP (1:1000; A6455, Invitrogen) and anti-PDF (1:5000; mAb C7, Developmental Study Hybridoma Bank, donated by Justin Blau), rabbit anti-DILP2, anti-sNPF recognizing the sNPF propeptide (for both 1:2000; Jan A. Veenstra [100,101]). The secondary antibodies used: Alexa Fluor 488 (goat anti-mouse, 1:250) Cy3 (goat anti-rabbit, 1:500) (Thermo Fisher Scientific), DyLight488 (goat-anti-rabbit 1:250) and DyLight649 (goat anti-mouse, 1:250) (Jackson ImmunoResearch, Dianova). Staining was visualized adopting either a semi-confocal (Nikon Eclipse 80i equipped with a QiCAM Fast Camera using the Image ProPlus software) or confocal microscope (ZEISS LSM700 running ZEN Lite software or Leica SP8).

### Live optical imaging in the insulin producing cells

To real-time monitor cAMP or Ca^2+^ concentration changes in the IPCs, live optical imaging was performed using the genetically encoded cAMP sensor, Epac1-camps [62] and the genetically encoded Ca^2+^ sensor GCaMP3.0 [82]. Relying on the UAS-GAL4 binary system [102], the sensors were expressed specifically in the IPCs (*dilp2(p)*>*Epac1-camps* or *dilp2(p)>GCaMP3*.*0*). Female flies, maintained at 25°C in LD 12:12, were anesthetized on ice before brain dissections in cold hemolymph-like saline (HL3 [103]) and mounted at the bottom of a cap of a plastic Petri dish (35x10 mm, Becton Dickenson Labware, New Jersey) in HL3 with the dorsal surface up. Brains were allowed to recover from dissection 15 min prior to imaging. Live imaging was conducted by using an epifluorescent imaging setup (VisiChrome High Speed Polychromator System, ZEISS Axioskop2 FS plus, Photometrics CoolSNAP HQ CCD camera, Visitron Systems GmbH) Visitron Systems GmbH) using a 40x dipping objective (ZEISS 40x/1.0 DIC VIS-IR). IPCs were brought into focus and regions of interest were defined on single cell bodies using the Visiview Software (version 2.1.1, Visitron Systems, Puchheim, Germany). Time-lapse frames were imaged with 0.2 Hz and 4x binning by exciting the CFP fluorophore of the cAMP sensor with 434/17 nm light or the GFP fluorescence of GCaMP3 with 488/10 nm light. For cAMP imaging, CFP and YFP emissions were separately detected using a Photometrics DualView2 beam splitter. After measuring baseline FRETs for ~100 s, substances were bath-applied drop-wise using a pipette, and imaging was performed for 1000 s. The neuropeptides used in this study were applied in a final concentration of 10 **μ**M in 0.1% DMSO in HL3. The water-soluble forskolin derivate NKH477 (10 **μ**M, Sigma Aldrich) or the cholinergic agonist carbamylcholine (1mM, Sigma Aldrich) served as positive controls in cAMP and Ca^2+^ imaging, respectively, while HL3 alone with 0.1% DMSO was used as negative control. In the case of tetrodotoxin (TTX) treatments, brains were incubated for 15 min in 2 **μ**M TTX in HL3 prior to imaging and substances were co-applied together with 2 **μ**M TTX. Inverse Fluorescence Resonance Energy Transfer (iFRET) was calculated over time according to the following equation: iFRET = CFP/(YFP-CFP*0.357) [62]. Thereby, raw CFP and YFP emission data were background corrected; in addition, YFP data were further corrected by subtracting the CFP spillover into the YFP signal, which was determined as 0.357 (35.7% of the CFP signal). Next, iFRET traces of individual neurons were normalized to baseline and were averaged for each treatment. Finally, maximum iFRET changes were calculated for individual neurons to quantify and contrast response amplitudes of the different treatments. The following synthetic neuropeptides were used in this study: pigment dispersing factor (PDF: *NSELINSLLSLPKNMNDAa*; Iris Biotech GmbH), short neuropeptide F-1 (sNPF-1: *AQRSPSLRLRFa*; Iris Biotech GmbH), adipokinetic hormone (AKH: *pQLTFSPDWa*, NovoPro Bioscience), allatostatin-C (Ast-C: *pEVRYRQCYFNPISCF*, gift from Paul H. Taghert), Tachykinin 4 (DTK-4: *APVNSFVGMRa*, gift from Paul H. Taghert), dFMRFamide 4 (SDNFMRFa, gift from Paul H. Taghert).

### Intracellular Ca^2+^ level

For Ca^2+^ imaging, brains expressed the GCamp3.0 sensor in the insulin producing cells (*dilp2(p)>GCaMP3.0*) [104]. The preparation of the brain samples was the same as in the case of cAMP imaging, and the same microscope was used with a modified setup, measuring GFP fluorescence without a beam splitter. The cholinergic agonist carbamylcholine (1 mM CCh) was used to generate rapid Ca^2+^ increases (Nakai et al. 2001). After subtraction of background fluorescence, changes in fluorescence intensity were calculated for each ROI as Δ(F/F_0_) = [(F_n_—F_0_)/F_0_] x 100 with F_n_ as fluorescence intensity at time point *n* and F_0_ as the baseline fluorescence calculated prior to the application of the different substances to the brain.

### PCR for *timeless* genotyping

To ensure genetic homogeneity for the *tim* locus, between the experimental flies and their corresponding controls, all the strains used in this study were genotyped in order to identify the *tim* allele present in their genome (summarized in S1 Table). The genomic DNA was extracted from individual adult females (10 flies per genotype) by homogenizing them in 50 **μ**l of extraction buffer (Tris HCl pH = 8.2 10 mM, EDTA 2 mM, NaCl 25 mM); after addition of 1 **μ**l of Proteinase K (10 mg/ml) samples were incubated at 37°C for 45 min, followed by 3 min at 100°C. The *tim* region containing the polymorphic site was amplified using a reverse primer *(5’-AGATTCCACAAGATCGTGTT-3’)* and two different forward primers (*ls-tim*: *5’-TGGAATAATCAGAACTTTGA-3’; s-tim*: *5’-TGGAATAATCAGAACTTTAT-3’)* that allow selective amplification of the different *tim* alleles [37].

### qRT-PCR assays

Larvae of the following genotypes: *Act>sNPFR1-RNAi*, *Act>+* and +>*sNPFR1-RNAi*, were reared under standard conditions at 23°C and LD12:12 until eclosion. Newly eclosed female flies were collected and subsequently exposed to low (12°C) or high (23°C) temperature and short photoperiod (8h:16hL:D) for 11 days. mRNA was isolated from whole bodies of 10 females. mRNA was reverse-transcribed with SuperScript II First-Strand Synthesis SuperMix (Invitrogen). PCRs were performed on a CFX96 Touch Real Time PCR Detector System (Bio Rad) with GoTaq qPCR Master Mix (Promega), using the following primers: *sNPFR- F*: *5′- CGACCATCAGATGCACCA -3′*, *R*: *5′-CGTCCGTCTCGTCTGTCC -3′; rp49 F*: *5′- ATCGGTTACGGATCGAACAA-3′*, *R*: *5′- GACAATCTCCTTGCGCTTCT-3′*. The results are shown as relative expression ratios obtained with the 2^-ΔΔCt^ method ± SEM. RP49 was used as reference. Results are shown in S2 Fig.

### Statistics

Data were analysed with R statistical software (version 3.0.1, www.r-project.org) and plotted using GraphPad Prism 6 software. In the case of normally distributed data (Shapiro-Wilk normality test, p>0.05), statistical significance was tested by one- or two-way ANOVA with *post-hoc* Tukey's HSD tests, while data that were not normally distributed were analyzed by Wilcoxon or Mann-Whitney test. In the case of multiple comparisons, raw p-values were further adjusted using Bonferroni correction, and these corrected p-values served as significance levels. When analyzing dormancy assays, all data were transformed to arcsine. For simplicity, figures in the *Results* section show untransformed data (dormancy, %).

## Supporting information

S1 Table The *timeless* background of the *Drosophila* strains used in this study, determined by genotyping PCR. *ls* = long and short allelic variant; s = short allelic variant.
(TIFF)

S2 Table Numerical data on which each graph was based, and pertinent statistical significance values. (XLSX)

S1 Fig Genetic manipulation of the dorsal-lateral peptidergic neurons has no effect on dormancy. (A) Hypersensitization of DLPs through the expression of a bacterial sodium channel (*Crz*_*1*_>*Na*^*+*^*ChBac*) does not alter quiescence levels (no difference from the Gal4 control). (B) Overexpression of sNPF in the DLPs does not influence the dormancy (no difference from the UAS control). Numbers within bars refer to the number of dissected females considered in the assays. Data are presented as mean ± SEM. ANOVA on arcsine transformations, followed by *post-hoc* Tukey HSD test. ***p<0.001, n.s. not significant.
(TIFF)

S2 Fig qRT-PCR of dsRNAi knockdown of *sNPFR1* at 23°C (A) and 12\C (B).
(TIFF)
