# Supplementary figures and images for: Peptidergic signaling from clock neurons regulates reproductive dormancy in Drosophila melanogaster
Source: PLoS Genet. 2019 Jun 13;15(6):e1008158. doi: 10.1371/journal.pgen.1008158 (PMC6592559; doi:10.1371/journal.pgen.1008158)

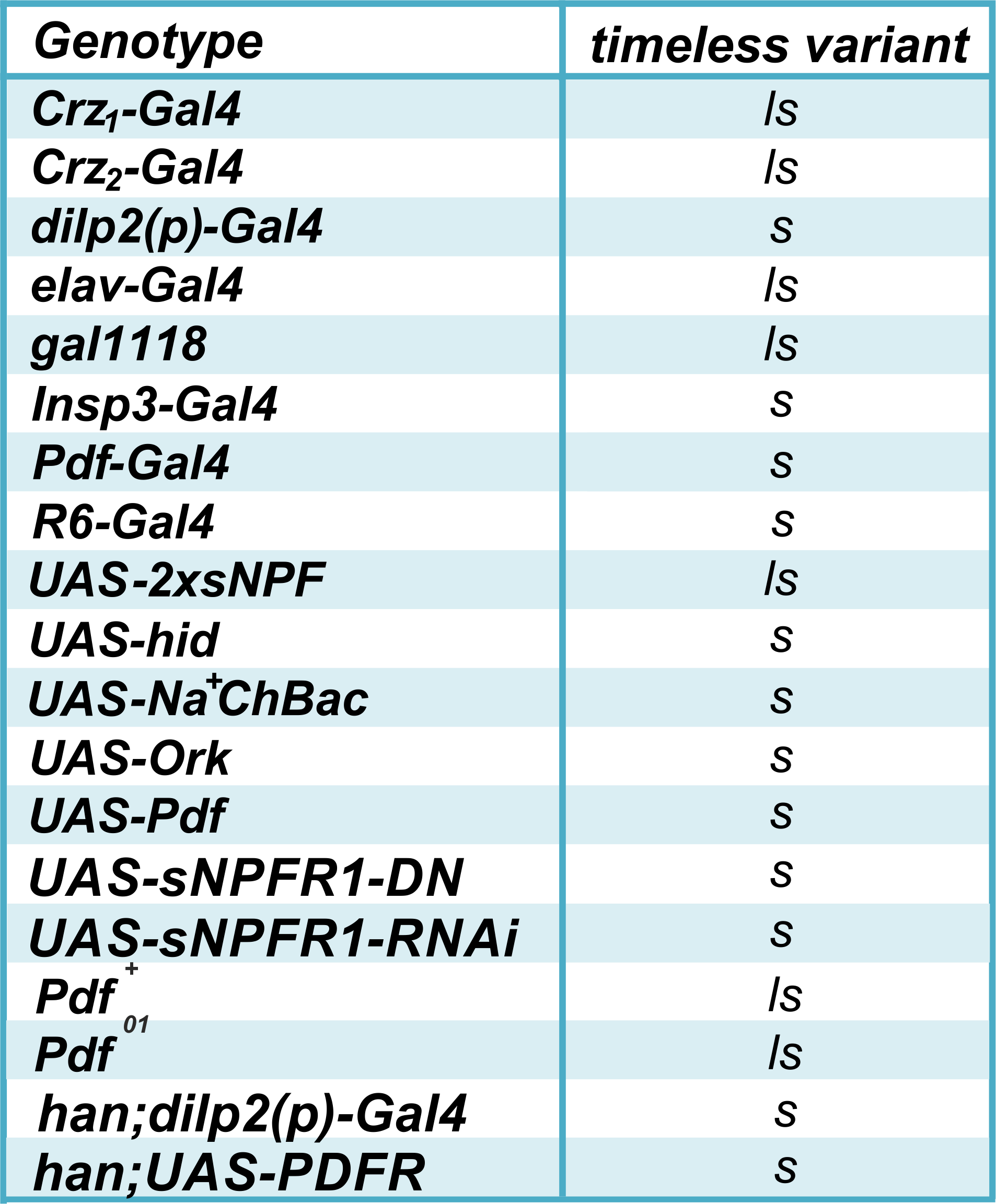

Supplement: S1 Table — ls = long and short allelic variant; s = short allelic variant. (TIFF) [file pgen.1008158.s001.tiff]

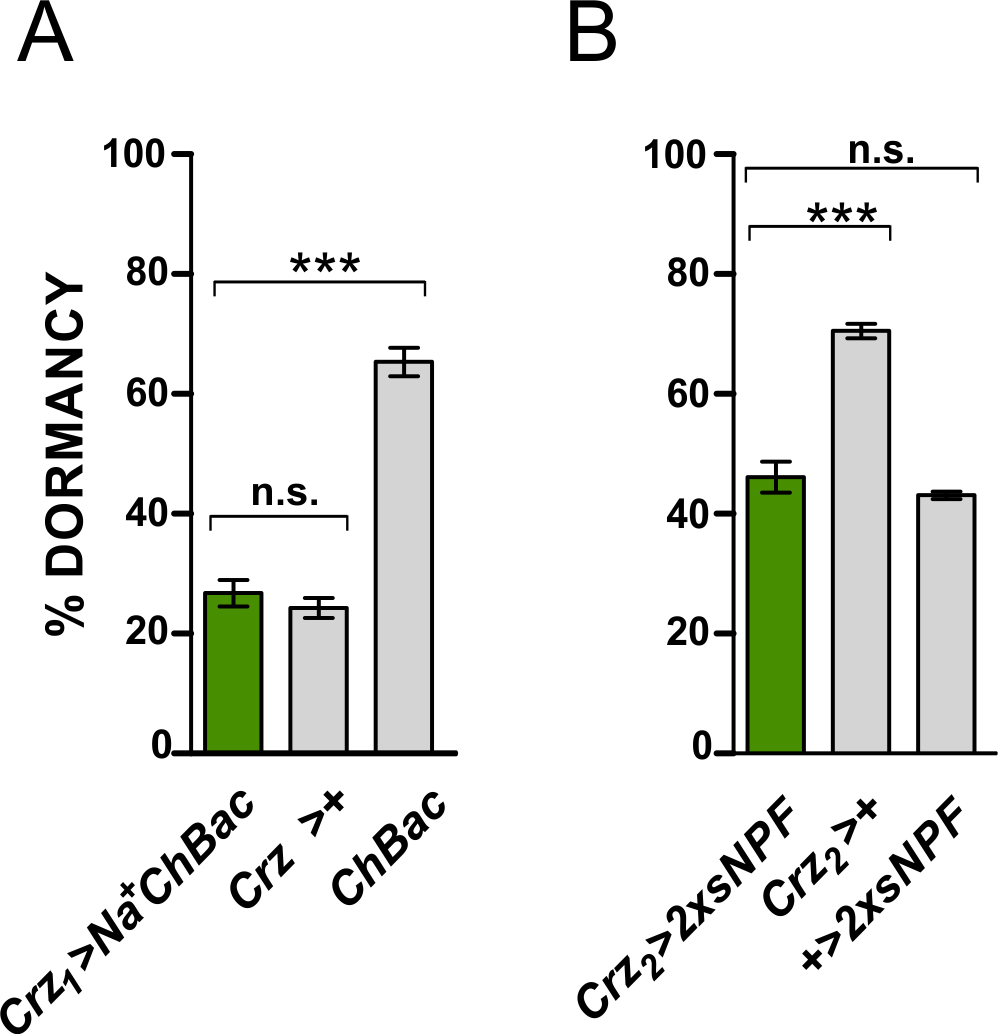

Supplement: S1 Fig — (A) Hypersensitization of DLPs through the expression of a bacterial sodium channel (Crz1>Na+ChBac) does not alter quiescence levels (no difference from the Gal4 control). (B) Overexpression of sNPF in the DLPs does not influence the dormancy (no difference from the UAS control). Numbers within bars refer to the number of dissected females considered in the assays. Data are presented as mean ± SEM. ANOVA on arcsine transformations, followed by post-hoc Tukey HSD test. ***p<0.001, n.s. not significant. (TIFF) [file pgen.1008158.s003.tiff]

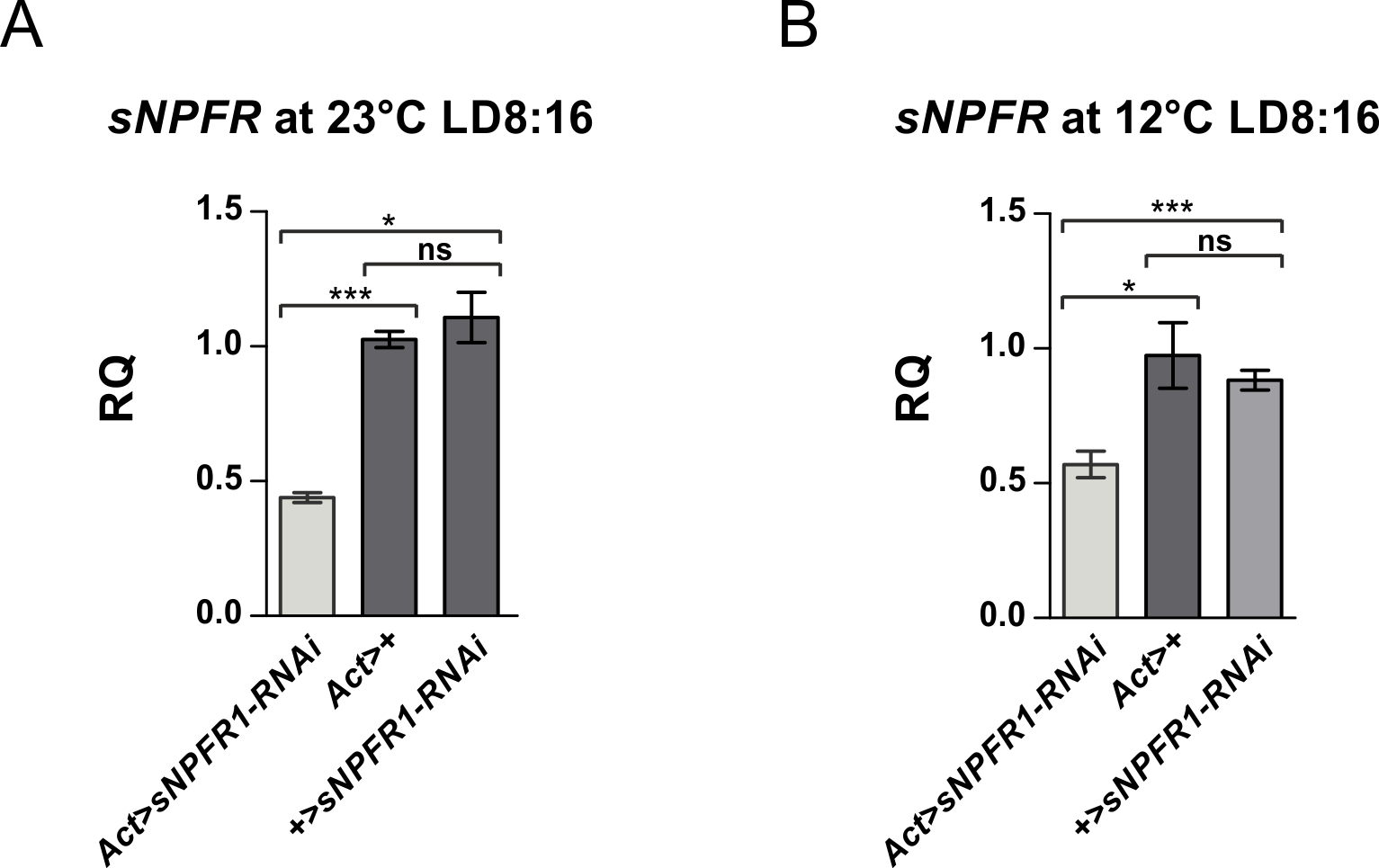

Supplement: S2 Fig — qRT-PCR of dsRNAi knockdown of sNPFR1 at 23°C (A) and 12\C (B). (TIFF) [file pgen.1008158.s004.tiff]
